# Family functioning and quality of parent-adolescent relationship: cross-sectional associations with adolescent weight-related behaviors and weight status

**DOI:** 10.1186/s12966-016-0393-7

**Published:** 2016-06-14

**Authors:** Jess Haines, Sheryl L. Rifas-Shiman, Nicholas J. Horton, Ken Kleinman, Katherine W. Bauer, Kirsten K. Davison, Kathryn Walton, S. Bryn Austin, Alison E. Field, Matthew W. Gillman

**Affiliations:** Department of Family Relations and Applied Nutrition, Room 226, Macdonald Stewart Hall, University of Guelph, 50 Stone Road East, Guelph, ON N1G 2 W1 Canada; Obesity Prevention Program, Department of Population Medicine, Harvard Medical School and Harvard Pilgrim Health Care Institute, Boston, USA; Department of Mathematics and Statistics, Amherst College, Amherst, USA; Department of Nutrition, Harvard T.H. Chan School of Public Health, Boston, USA; Division of Adolescent and Young Adult Medicine, Boston Children’s Hospital, Boston, USA; Channing Division of Network Medicine, Brigham and Women’s Hospital and Harvard Medical School, Boston, USA; Department of Social and Behavioral Sciences, Harvard T.H. Chan School of Public Health, Boston, USA; Department of Epidemiology, Harvard T.H. Chan School of Public Health, Boston, USA; Department of Nutritional Sciences, University of Michigan School of Public Health, Ann Arbor, USA; Department of Biostatistics and Epidemiology, University of Massachusetts Amherst School of Public Health and Health Sciences, Amherst, USA

**Keywords:** Family functioning, Weight-related behaviors, Parent-adolescent relationship, Obesity risk

## Abstract

**Background:**

Little is known about how factors within the general family environment are associated with weight and related behaviors among adolescents/young adults.

**Methods:**

We studied 3768 females and 2614 males, 14–24 years old in 2011, participating in the Growing Up Today Study 2. We used generalized mixed models to examine cross-sectional associations of family functioning and quality of mother- and father-adolescent relationship with adolescent/young adult weight status, disordered eating, intake of fast food and sugar-sweetened beverages, screen time, physical activity, and sleep duration. In all models, we included participant’s age and family structure.

**Results:**

Eighty percent of participants reported high family functioning and 60 % and 50 % of participants reported high-quality mother and father relationship, respectively. Among both males and females, high family functioning was associated with lower odds of disordered eating (adjusted odds ratio [AOR] females = 0.53; 95 % Confidence Interval [CI] = 0.45–0.63; AOR males = 0.48; CI = 0.39–0.60), insufficient physical activity, i.e., less than 1 h/day, (AOR females = 0.74; CI = 0.61–0.89; AOR males = 0.73; CI = 0.58–0.92), and insufficient sleep, i.e., less than 7 h/day, (AOR females = 0.56; CI = 0.45–0.68; AOR males = 0.65; CI 0.5–0.85). High family functioning was also associated with lower odds of being overweight/obese (AOR = 0.73; CI = 0.60–0.88) and eating fast food one or more times/week (AOR = 0.74; CI = 0.61–0.89) among females only. Among females, high-quality mother and father relationship were both associated with lower odds of being overweight/obese and disordered eating, eating fast food, and insufficient sleep and the magnitude of associations were similar for mother and father relationship quality (AOR range 0.61–0.84). Among males, high-quality mother and father relationship were both associated with lower odds of disordered eating, insufficient physical activity and insufficient sleep, but only father relationship quality was associated with lower odds of overweight/obesity.

**Conclusions:**

Adolescents/young adults reporting high family functioning and more positive relationships with their parents reported better weight-related behaviors. For weight status, females appear to be affected equally by the quality of their relationship with both parents, whereas males may be more affected by their relationship with fathers.

## Background

Currently, the majority of adolescents and young adults in the United States are falling short of recommended dietary and physical activity behavioral goals. For example, less than 8 % of adolescents and 5 % of young adults meet the recommended minimum of 60 min per day of physical activity [1]; while 16 % of adolescents and 20 % of young adults have been found to have high intakes of sugar-sweetened beverage intake (≥500 kcal/day) [2]. Disordered eating (e.g., fasting, purging and binge eating) [6] and insufficient sleep (less than 7 h/night) are also common among adolescents and young adults [3]; these behaviors contribute alongside poor dietary quality and low physical activity levels to excessive weight gain and a high incidence of obesity during these life stages [4–7]. Obesity during adolescence and early adulthood is associated with increased risk of obesity in later adulthood [8], as well as increased risk of chronic health conditions such as cardiovascular disease, type 2 diabetes and many forms of cancer [9, 10]. Dietary, disordered eating, and physical activity behaviors have been found to track between adolescence/early adulthood and later adulthood [11, 12], suggesting that understanding the key determinants of obesity and related behaviors during these critical life stages is needed to identify appropriate health promotion strategies.

The family environment serves a fundamental role in the establishment of dietary, physical activity, and other weight-related behaviors among youth. While a number of studies have explored how diet- or activity-related parental behaviors, including feeding practices [13–15], modeling [16, 17] and providing support for health behaviors [18, 19], as well as general parenting style [20–22] may influence adolescent weight and related behaviors, few studies have explored how other factors in the general family environment, such as family functioning or quality of parent-adolescent relationship, may influence these outcomes. Family systems theory asserts that an individual’s behaviors must be understood within the family context [23]. These general family factors reflect the overarching family context within which adolescents develop; thus, these factors may be powerful determinants of weight and related behaviors. Given that adolescence and young adulthood are characterized by increasing independence from family where peer and other non-family factors may have increased influence on behavior, it is particularly important to understand to what degree these general family factors may still provide a foundation for behavior as youth move through adolescence. Understanding how these general family factors are associated with weight and related behaviors can help inform family-based obesity prevention interventions.

Family functioning is a complex phenomenon describing structural and organizational properties of a family group and the patterns of interactions between the group’s members [24]. Specifically, it describes how families manage their daily routines, fulfill their roles within the family, and communicate and connect emotionally [25]. Although research has shown that adolescents with eating disorders report higher levels of family dysfunction [26, 27], only a small number of studies have examined whether family functioning is associated with adolescent obesity [28–31]. Results of this existing research are inconsistent; two studies found no significant association between family functioning [30, 31] and adolescent weight status and two found that higher family dysfunction was associated with higher weight status among youth [18, 19]. The majority of research exploring the association between family functioning and obesity risk has included small, convenience samples [28, 30, 31]. A few studies have also explored how single aspects of family functioning, i.e., emotional connection and family conflict, are associated with obesity risk [32], eating behavior [33] and physical activity among youth [34]. Only one study has explored associations between overall family functioning and obesity-related behaviors among adolescents in the United States [29]. Berge and colleagues found that higher family functioning was associated with more healthful dietary intake, less sedentary behavior and greater physical activity in their sample of 2793 Minnesota youth [29]. Our research builds on this existing research by examining the associations between family functioning and obesity and related behaviors in a large, nationwide sample of adolescents/young adults in the United States.

In addition to the overall functioning of the family unit, the quality of the unique relationship between a parent and his/her adolescent/young adult child may influence adolescent weight status and related behaviors. The quality of the parent-adolescent relationship has been explored as the level of attachment, bonding and emotional connection, and communication and in the relationship [32]. Limited research has explored how parent-adolescent relationship quality is associated with adolescent obesity [35, 36], dietary intake [37] and physical activity [34], however, the majority have found positive associations between quality of the parent-adolescent relationship and more healthful outcomes. Existing research exploring these associations has combined information across parents without distinguishing fathers from mothers or has examined only mother-adolescent relationship quality. Thus, little is known about how father-adolescent relationship quality is associated with adolescent weight status and weight-related behaviors.

The objective of this study was to examine the extent to which family functioning and mother- and father-adolescent relationship quality are associated with weight status and weight-related behaviours, i.e., disordered eating, intake of fast food and sugar sweetened beverages, screen time, physical activity, and sleep duration, among a large, nationwide sample of adolescents/young adults in the United States. We hypothesized that high family functioning and high quality mother-adolescent and father-adolescent relationship would be associated with less obesity/overweight weight status and more healthful weight-related behaviors.

## Methods

### Study population

Growing Up Today Study 2 (GUTS 2) is a longitudinal cohort study that was established in 2004 to assess associations of diet and activity to height velocity and weight gain among a nationwide sample of adolescents in the United States. Participants are offspring of women taking part in the Nurses’ Health Study II (NHS II). To recruit the participants in the GUTS 2 cohort, we mailed letters to 20,700 women in NHS II who had children aged 9–17 years, which described the purpose of the study and requested permission for their child to participate. In 2004, we mailed questionnaires to 8826 females and 8454 males whose mothers granted consent. A total of 6002 girls and 4918 boys returned completed questionnaires, thereby assenting to participate. Follow-up questionnaires (on-line and mailed paper copies) were sent in 2006, 2008, and 2011.

For this study, we restricted analyses to participants who responded to the 2011 questionnaire, when family functioning and quality of parental-adolescent relationship were assessed (*n* = 6659). Participants missing data on family functioning and quality of parental-adolescent relationship were excluded from the analyses (*n* = 277), resulting in an analytic sample of 6382 (3768 females and 2614 males). In the analytic sample, 21 % had at least one sibling in the cohort (5013 unique families). The study was approved by the Partners Human Research Committee.

### Measures

#### Exposure variables

##### Family functioning.

Participants reported their level of family functioning using 9 items from the 12-item General Functioning Scale of the Family Assessment Device [24]. To meet space limitations on the survey, we did not include similarly worded items for 3 of the items (e.g., we included “individuals are accepted for who they are” but did not include “we feel accepted for who we are”). The items included on the General Functioning Scale measure the overall health/pathology of the family relating to six dimensions of family functioning: a) problem solving, b) communication, c) roles, d) affective responsiveness, e) affective involvement, and f) behavioral control. The scale consists of statements about families to which adolescents indicated the degree to which they agree on a 4-point scale (strongly disagree to strongly agree) [13, 33]. We divided the sum by 9 to give a mean score from 1.0 to 4.0 with lower scores implying better family functioning. We used the cut-point ≤ 2.17 as high functioning based on previous evidence that this cut-point effectively discriminates between healthy and unhealthy functioning in families with young children and older adolescents [38–40].

##### Quality of mother-adolescent and father-adolescent relationship.

Participants reported separately their degree of satisfaction with respect to conflict resolution, emotional support, time spent together, and communication with their mothers and fathers using a 9-item parent-adolescent relationship satisfaction scale [41]; sample question: “I am satisfied with the amount of time my parent and I spend together”. The scale was developed for research in adolescents and has been shown to be associated with other health behaviors among youth [41].

Participants were able to opt out of the scale regarding either parent if the scale was not applicable; 3044 (81 %) females and 1996 (76 %) males, provided information on both mother and father-adolescent relationship quality. Items were rated on a 5-point Likert scale (strongly disagree to strongly agree) and we divided the sum by 9 to give a mean score ranging from 1.0 to 5.0 with a higher score representing higher quality of the parent-adolescent relationship. No established cut-points exist for this scale. We dichotomized mean scores into high and low quality relationship using the cut-point ≥ 4 for high quality relationship based on a priori classification that agreement with most items would suggest a high relationship quality.

#### Outcome variables

##### Disordered eating behaviors.

Participants who reported that they were currently trying to lose weight were asked if they engaged in any of the following behaviors: vomiting, using laxatives, fasting, or binge eating behaviors using items found to be valid compared with interviews [42, 43]. Participants who answered affirmatively to any of these questions were classified as engaging in disordered eating behaviors.

##### Screen time.

Participants self-reported their television viewing and other screen time with a question reading: “On average, how many hours per week do you spend in each of the following activities?” which then lists four categories of screen time: television, videos, computer games, and Internet [44]. We summed the average hours across these four categories, divided by seven to get the average daily hours and dichotomized hours of screen time as ≥ 2 h or < 2 h/day based on recommendations from the American Academy of Pediatrics [45].

##### Physical activity.

Participants self-reported their level of physical activity with the Youth/Adolescent Activity Questionnaire, which is based on the validated self-administered physical activity assessment tool developed for NHS II [46]. We dichotomized physical activity as ≥ 1 h or < 1 h of activity/day based on recommendations from the Centers for Disease Control and Prevention [47].

##### Sleep duration.

Participants self-reported their sleep duration with an item reading: “In a typical 24 h, how many hours of sleep do you get?” Eight response options ranged from “less than 5 h” to “11 or more hours.” We categorized sleep duration as < 7 h/day, 7–9 h/day (referent), or > 9 h/day based on recommendations from the National Sleep Foundation [48]. However, due to the small number of participants in the > 9 h category, we collapsed the 7–9 h/day and > 9 h/day into a single category.

##### Fast food intake.

Participants self-reported their frequency of eating foods from a fast food restaurant (i.e. McDonalds, KFC and Wendy’s). We dichotomized frequency of fast food intake as ≥ 1 servings/week or < 1 serving/week [49, 50].

##### Sugar sweetened beverage intake.

Participants self-reported their intake of sugar sweetened beverages, including soda and fruit drinks, using items from the Youth/Adolescent Food Frequency Questionnaire [51]. We dichotomized servings of sugar sweetened beverages as ≥ 1 servings/day or < 1 servings/day [52].

##### Weight status.

Participants self-reported their height and weight, which we used to calculate body mass index (BMI). We dichotomized weight status as overweight/obese or normal weight based on the International Obesity Task Force cut-points [50]. According to these cut-offs, males and females were considered overweight/obese at the 90.5 and 89.3 percentiles, respectively [53].

#### Covariates

##### Age.

We calculated participants’ ages from their birthdate and the date that the 2011 questionnaire was returned.

##### Family structure.

We determined family structure based on mothers’ most recent report of living arrangement, which was the 2005 NHS II questionnaire. Response options included: alone, with spouse or partner, with other adult family member, and other. Mothers were asked to identify all relevant responses. We coded family structure as: Mother lives with spouse/partner and Other, which included alone, with other adult family member, and other.

### Statistical analyses

We examined the correlations between family functioning, mother-adolescent relationship quality and father-adolescent relationship quality using the phi coefficient. The phi coefficient is similar in its interpretation to the Pearson correlation coefficient, but it is a measure of correlation between two binary variables [54]. To adjust for covariates and to account for correlation between siblings (5013 unique families), we used generalized mixed models (Proc GLIMMIX) to model associations between family functioning and quality of mother- and father-adolescent relationship and adolescent weight status and weight-related behaviors. We fit separate models for each of the outcome variables. All models adjusted for adolescent’s age and family structure. We also fit models that additionally adjusted for mothers' BMI at age 18 years, adolescent living situation (with parents or other), and adolescent race/ethnicity; the effect estimate remained virtually unchanged (results not shown), so we report only estimates from the more parsimonious model. To provide a point of reference for the odds ratio estimates, the predictive prevalence for each outcome variable was calculated for those with high and low family functioning and for those with high and low parent-adolescent relationship quality. We also fit models that included interaction terms (e.g., family functioning * family structure) to examine whether the associations between these general family factors (i.e., family functioning, mother-adolescent relationship quality, or father-adolescent relationship quality) and adolescent weight-related outcomes and behaviors differed by family structure. We found limited evidence of interaction by family structure (results not shown), so report only results from the main effect model. Due to known differences in weight status and weight-related behaviors by sex, all analyses were stratified by sex. We conducted all analyses with SAS software (version 9.3 SAS Institute, Cary, North Carolina, USA).

## Results

### Participant characteristics

Participants were between the ages of 14–24 years; the mean age was 20 years (Table 1). The majority (92.4 %) of the participants identified as ‘white’. Nearly 80 % of both male and female participants reported a high level of family functioning. A high-quality relationship with mother was reported by 64 % of females and 60 % of males. High-quality father relationship was reported by 50 % of females and 52 % of males (Table 1).

**Table 1:** Characteristics of adolescents in the Growing Up Today Study 2, stratified by sex

	Females, *N* = 3768		Males, *N* = 2614	
	Non-missing values *N*	*N* (%)	Non-missing values *N*	*N *(%)
Age	3768		2614	
Years, mean (SD)		20.4 (1.8)		20.2 (1.9)
Family Structure	3678		2572	
Mother lives with spouse/partner		2920 (79.4)		2071 (80.5)
High Family Functioning	3766		2608	
General Functioning score ≤ 2.17		2990 (79.4)		2069 (79.3)
High Quality Mother-Adolescent Relationship	3139		2029	
Relationship satisfaction score ≥ 4		2004 (64.1)		1217 (60.0)
High Quality Father-Adolescent Relationship	3056		2008	
Relationship satisfaction score ≥ 4		1525 (49.9)		1044 (52.0)
Overweight/Obese, IOTF cut-points	3728		2600	
Males: 90.5 % tile Females: 89.3 %tile		847 (22.7)		834 (32.1)
Disordered Eating,	3732		2390	
Engaged in vomiting and/or laxatives, and/or fasting and/or binge eating		1252 (33.5)		598 (23.1)
Fast Food from a fast food restaurant (McDonalds, KFC, Wendy’s etc.)	3665		2540	
≥ 1 serving/week		921 (25.1)		1075 (42.3)
Sugar Sweetened Beverages,	3680		2545	
≥ 1serving/day		480 (13.0)		894 (35.1)
Screen Time	3050		1988	
≥ 2 h/day		1028 (33.7)		873 (43.9)
Physical Activity	3101		2013	
< 1 h/day		1649 (53.2)		856 (42.5)
Sleep duration	3125		2036	
< 7 h/day		639 (20.4)		422 (20.7)

### Correlation between family functioning, mother-, and father-adolescent relationship quality

The correlation between family functioning and mother-adolescent relationship quality was *r* = 0.41 among both females and males. The correlation between family functioning and quality of father-adolescent relationship was *r* = 0.35 among females and *r* = 0.36 among males. The correlation between mother and father-relationship quality was *r* = 0.51 among females and *r* = 0.61 among males.

### Family functioning and adolescent weight status and weight-related behaviors

#### Females

**Fig. 1:**
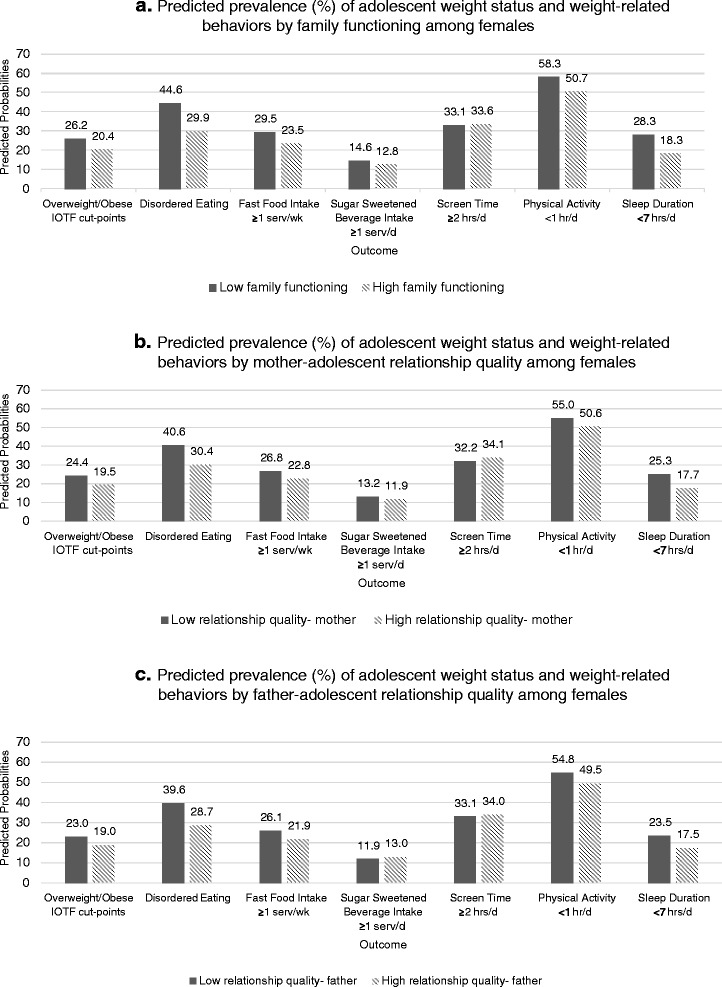
(a, b, and c) Predicted prevalence (%) of adolescent weight status and weight-related behaviors by general family environment among female participants in the Growing Up Today Study 2

Among females, higher family functioning was associated with lower odds of being overweight or obese (AOR = 0.73; 95 % CI 0.60, 0.88; Table 2). The predicted prevalence of overweight or obesity was 20 % among females with high family functioning, and 26 % for those with low family functioning (Fig. 1a). Higher family functioning was also associated with lower odds of engaging in disordered eating behaviors (AOR = 0.53; 95 % CI 0.45, 0.63), of eating fast food at least once/week (AOR = 0.74; 95 % CI 0.61, 0.89), of getting less than 1 h of physical activity/day (AOR = 0.74; 95 % CI 0.61, 0.89), and of sleeping less than 7 h/day (AOR = 0.57; 95 % CI 0.46, 0.70). We did not find associations between level of family functioning and either intake of sugar sweetened beverages or screen time among females (Table 2).

**Table 2:** Associations of general family environment with adolescent weight-related behaviours and overweight status among participants in the Growing Up Today Study 2, stratified by sex

	Overweight/obese IOTF cut-points males: 90.5 % tile females: 89.3 % tile	Disordered eating engaged in vomiting and/or laxatives, and/or fasting and/or binge eating	Fast food intake	Sugar sweetened beverage intake	Screen time	Physical activity	Sleep duration
			≥1 serv/week	≥1 serv/day	≥2 h/day	<1 h/day	<7 h/day
	OR^b^ (95 % CI)	OR^b^ (95 % CI)	OR^b^ (95 % CI)	OR^b^ (95 % CI)	OR^b^ (95 % CI)	OR^b^ (95 % CI)	OR^b^ (95 % CI)
Females (*N* = 3,768^a^)							
High Family Functioning	**0.73 (0.60,0.88)**	**0.53 (0.45, 0.63)**	**0.74 (0.61, 0.89)**	0.86 (0.68, 1.08)	1.03 (0.84, 1.24)	**0.74 (0.61, 0.89)**	**0.57 (0.46, 0.70)**
High Mother-Adolescent Relationship Quality	**0.75 (0.63, 0.91)**	**0.64 (0.54, 0.75)**	**0.81 (0.68, 0.97)**	0.89 (0.71, 1.12)	1.09 (0.93, 1.29)	**0.84 (0.72, 0.98)**	**0.64 (0.53, 0.77)**
High Father-Adolescent Relationship Quality	**0.79 (0.66, 0.94)**	**0.61 (0.52, 0.72)**	**0.80 (0.67, 0.95)**	1.10 (0.88, 1.38)	1.04 (0.89, 1.22)	**0.81 (0.70, 0.94)**	**0.69 (0.57, 0.82)**
Males (*N* = 2,614^a^)							
High Family Functioning	0.92 (0.75,1.14)	**0.48 (0.39, 0.60)**	1.01 (0.82, 1.24)	0.97 (0.78, 1.19)	0.87 (0.69, 1.09)	**0.73 (0.58, 0.92)**	**0.65 (0.50,0.85)**
High Mother-Adolescent Relationship Quality	1.04 (0.85,1.27)	**0.62 (0.51, 0.77)**	1.05 (0.86, 1.26)	1.09 (0.90, 1.33)	0.91 (0.75, 1.10	**0.78 (0.64, 0.94)**	**0.63 (0.51,0.79)**
High Father-Adolescent Relationship Quality	**0.80 (0.66,0.98)**	**0.72 (0.58, 0.89)**	0.83 (0.69, 1.00)	1.02 (0.84, 1.24)	0.98 (0.82, 1.19)	**0.69 (0.57, 0.83)**	**0.61 (0.49,0.77)**

^a^Numbers differ slightly due to missing data in the outcome measures

^b^All odds ratios adjusted for family structure and adolescent age

When 95 % CI does not include 1.0, the estimate and 95 % CI are bolded

#### Males

Among males, high family functioning was associated with lower odds of engaging in disordered eating behaviors (AOR = 0.48; 95 % CI 0.39, 0.60; Table 2) of getting less than 1 h of physical activity/day (AOR = 0.73; 95 % CI 0.58, 0.92) and of sleeping less than 7 h/day (AOR = 0.65; 95 % CI 0.50, 0.85). Level of family functioning was not associated with weight status, intake of fast food, intake of sugar sweetened beverages, or screen time among males (Table 2).

### Mother and father relationship quality and adolescent weight status and weight-related behaviors

#### Females

Among females, high-quality mother relationship was associated with lower odds of being overweight or obese (AOR = 0.75; 95 % CI 0.63, 0.91). The predicted prevalence of overweight or obesity was 19 % among females with high-quality mother-adolescent relationship and 24 % for those with low-quality mother relationship (Fig. 1b). High-quality mother relationship was also associated with lower odds of engaging in disordered eating behaviors (AOR = 0.64; 95 % CI 0.54, 2.22), of eating fast food at least once/week (AOR = 0.81; 95 % CI 0.68, 0.97), of getting less than 1 h of physical activity/day (AOR = 0.84; 95 % CI 0.72, 0.98) and of sleeping less than 7 h/day (AOR = 0.64; 95 % CI 0.53, 0.77). We did not find an association between mother relationship quality and intake of sugar sweetened beverages or screen time among females. Results for father relationship quality were similar in direction and magnitude as those seen for mother relationship quality (Table 2).

#### Males

Among males high-quality mother relationship was associated with lower odds of engaging in disordered eating behaviors (AOR = 0.62; 95 % CI 0.51, 0.77; Table 2), of getting less than 1 h of physical activity/day (AOR = 0.78; 95 % CI 0.64, 0.94), and of sleeping less than 7 h/day (AOR = 0.63; 95 % CI 0.51, 0.79). Mother relationship quality was not associated with weight status, intake of fast food, intake of sugar sweetened beverages, or screen time among males (Table 2).

**Fig. 2:**
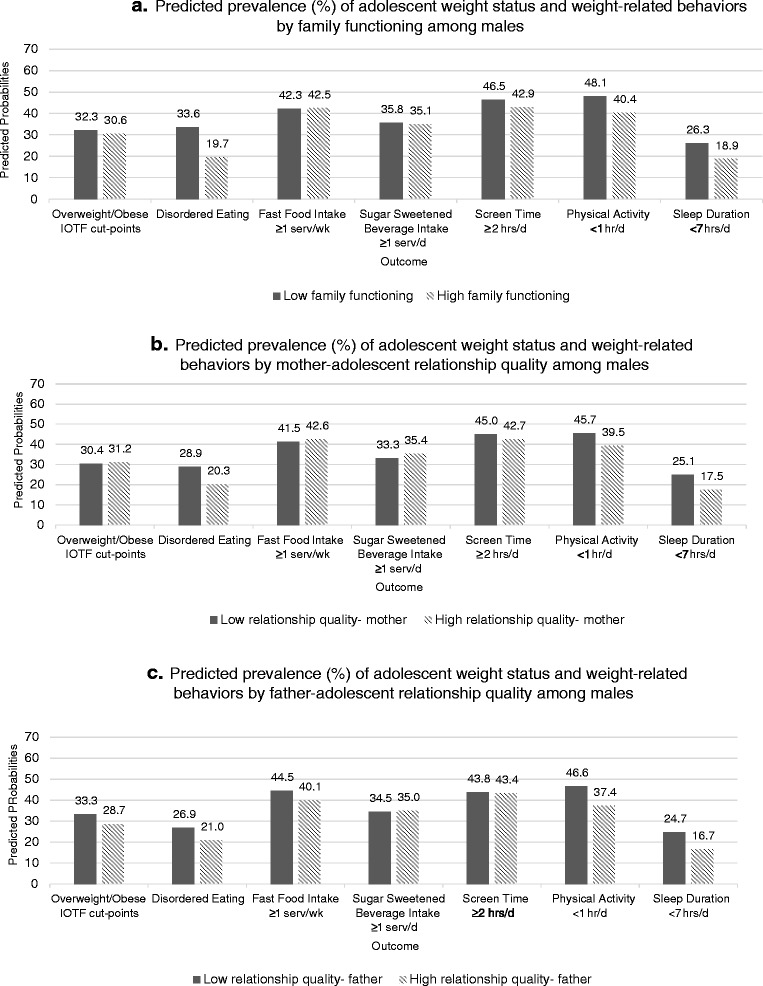
(a, b, and c) Predicted prevalence (%) of adolescent weight status and weight-related behaviors by general family environment among male participants in the Growing Up Today Study 2

High-quality father relationship was associated with lower odds of being overweight or obese (AOR = 0.80; 95 % CI 0.66, 0.98). The predicted prevalence of overweight or obesity was 26 % among males with high-quality father-adolescent relationship and 33 % for those with low-quality father relationship (Fig. 2c). High-quality father relationship was also associated with lower odds of engaging in disordered eating behaviors (AOR = 0.72; 95 % CI 0.58, 0.89), of eating fast food at least once/week (0.83; 95 % CI 0.69, 1.00), of getting less than 1 h of physical activity/day (AOR = 0.69; 95 % CI 0.57, 0.83), and of sleeping less than 7 h/day (AOR = 0.61; 95 % CI 0.49, 0.77). We did not find an association between father relationship quality and intake of sugar-sweetened beverages or screen time among males (Table 2).

## Discussion

In this large, nationwide cohort of adolescents/young adults, we observed that adolescents/young adults from families with high family functioning and high-quality of mother- and father-adolescent relationships were less likely to have overweight or obesity and less likely to engage in unhealthful weight-related behaviors, although the pattern of these associations differed by sex. Associations between family functioning and weight-related behaviors were similar for males and females; however, family functioning was associated with weight status among females only. Both males and females who reported positive relationships with their parents reported better weight-related behaviors. Females were affected equally by the quality of their relationship with their mothers and fathers, whereas for males, weight status may be more affected by their relationship with fathers.

Our results are consistent with previous studies with smaller samples, which found that adolescents from families with high family functioning are less likely to engage in disordered eating behaviors [15, 16]. Epidemiologic studies exploring associations between family functioning and adolescent weight are few in number [28–31] and our results extend this research by examining this association in a large, nationwide sample. Similar to our results, in their sample of ethnically-diverse Minnesota youth, Berge and colleagues [29] found that while higher family functioning was associated with healthier eating and activity behaviors among both males and females, higher family functioning was associated with lower prevalence of overweight/obesity among females only. While family dysfunction may interfere with healthful behavior for both males and females due to the families’ limited ability to organize and plan routines related to these behaviors, the stronger association between family function and weight among females may be due to stronger reactivity and response to stress among females. Results from laboratory research suggest that, when exposed to similar stressors, adolescent females have stronger physiologic reactivity to stress than adolescent males [55]; this physiologic reactivity can lead to metabolic disturbances that can impair sensitization of satiety signals [56, 57]. Epidemiologic [58] and laboratory research [59] has also found that females engage in more disinhibited eating in response to stress than males, which could lead to greater weight gain among females. Elucidating the mechanisms regarding how factors in the general family environment influence weight outcomes among adolescents/young adults is an important area for future research.

Although our measure of mother- and father-adolescent relationship quality assessed adolescent satisfaction with a range of aspects of the parent-adolescent relationship, including closeness, conflict resolution, emotional support, time spent together, and communication, our results are similar to the majority of studies that have examined specific aspects of the parent relationship. For example, using data from 977 participants in the Study of Early Child Care and Youth Development, Anderson and colleagues [36] found that lower maternal attachment and sensitivity, assessed objectively during the preschool years, were associated with higher prevalence of obesity during adolescence. Insecure parent attachment in childhood was also found to be associated with greater intake of high calorie food among children and adults [37]. Level of bonding or closeness with a parent has also been shown to moderate the association between maternal-BMI and daughter-BMI [17–60] and parental and adolescent weight-related behaviors [17–61]. In contrast, using data from the National Longitudinal Study of Adolescent Health, Crossman and colleagues [35] found that a higher degree of closeness with a parent was associated with greater risk for excess weight gain among males, but not females. Our study builds on these findings by using a broad definition of parent-adolescent relationship that includes numerous aspects of the relationship and by providing insight into how the father-adolescent relationship may be associated with adolescent weight and related behaviors, which was not explored in these existing studies.

In our study, we found that among females, high quality father-adolescent relationship was associated with healthful adolescent weight and weight-related behaviors in a similar manner to the more frequently researched mother relationship. Among males, however, the quality of the father-adolescent relationship had a stronger relationship with weight status than did mother-adolescent relationship quality. Although research has demonstrated that fathers’ weight-related behaviors are associated with adolescent diet [17–62] and activity behaviors [63], it is possible that the same-sex parent relationship has a stronger influence on sons than daughters. Additional etiologic and intervention research involving fathers is needed to identify mechanisms by which father-relationship quality influences weight status in youth and to explore possible differences in these mechanisms by child sex.

A number of studies have also examined how parenting style is associated with adolescent weight and related behaviors [20–22, 25]. Parenting style is typically defined by level of parental warmth and control with four key parenting styles examined: authoritative, authoritarian, permissive and neglectful [25]. The majority of studies show that authoritative parenting, characterized by high warmth and high control, is associated with lower obesity risk and healthier weight-related behaviors among adolescents [64]. While adolescents with authoritative parents have been found to report higher overall life satisfaction [65], it is also possible that an authoritative parenting style may be associated with increased satisfaction with their relationship with their parents, which may be associated with healthier weight-related behaviors and outcomes. Taken together, this research suggests that the parent-adolescent relationship may be an important determinant of adolescent weight and related behaviors. Future research is needed to determine which aspects of the parent-adolescent relationship are the key determinants of adolescent behavior as well as the specific pathways by which these relationships influence adolescent behavior.

With the exception of obesity/overweight among males, which was only significantly associated with father-adolescent relationship quality, we found that family functioning and parent-adolescent relationship quality had similar associations across the weight-related behaviors and outcomes among both males and females. While the correlations of these exposure variables suggest they are somewhat distinct general family constructs, our results suggest that family functioning and parent-adolescent relationship quality may have similar influences on weight and related outcomes. However, for all outcomes (again, except for obesity among males), the association was stronger for family functioning, which may suggest it may be a stronger determinant of weight and related behaviors. Additional research is needed to confirm our findings and to explore how associations between general family factors and weight and related outcomes differ by sex.

This study had several limitations that should be considered when interpreting our results. As in all observational studies, residual and unmeasured confounding could be present. However, adjustment for potential socio-demographic confounders we did assess, including family structure and race/ethnicity did not have a substantive influence on our estimates of association. The data used to identify adolescents’ family structure was collected in 2005 and thus may not have been an accurate representation of adolescents’ family structure when the predictor and outcome measures for this analysis were assessed. Our analyses are cross-sectional; thus, we are unable to rule out reverse causation in the observed associations. However, this limitation applies mainly to the outcome of disordered eating as reverse causation is unlikely with the other outcomes explored. All measures were collected by self-report and individuals with higher BMIs are more likely to under-report their weight and dietary intake and over-report their physical activity. This reporting bias would most likely bias estimates towards the null. We calculated 42 tests and did not adjust for multiple comparisons. Of these tests, 25 were statistically significant at the 0.05 level, much larger than the 2 we would expect by chance. Although study participants reside throughout the US, our cohort is not a representative sample of US adolescents. Participants are children of registered nurses and the cohort is > 90 % white, which may reduce the generalizability of our findings.

## Conclusion

To date, few obesity prevention interventions have successfully changed dietary intake or physical activity among adolescents and fewer yet have limited weight gain [66]. Our results suggest that factors within the general family environment, including family functioning and quality of parent-adolescent relationship, may be important factors to address within interventions design to support healthful behaviors and weight status among adolescents. Future research should employ a longitudinal design to help elucidate the temporal and potentially bi-directional nature of the associations we found. Experimental research, such as family-based psychotherapy interventions, could also examine the extent to which improving family functioning or parent-child relations results in improved weight outcomes among adolescents.

## Abbreviations

AOR, adjusted odds ratio; BMI, body mass index; CI, confidence interval; GUTS, growing up today study; NHS, Nurses health study
